# Vitamin D in the prevention of left ventricular remodelling after an acute myocardial infarction: a randomized clinical trial

**DOI:** 10.1038/s41598-026-45330-6

**Published:** 2026-04-06

**Authors:** José Tuñón, Carlos Rodríguez-López, Jorge Balaguer, Sandra Gómez-Talavera, Antonio Rojas, Jorge Salamanca, Cecilia López García, María Asunción Esteve-Pastor, Ana Laffond, Cristian Herrera, Juan M. Escudier-Villa, Miguel Orejas, Marta Tomás, María López Álvarez, Mireia Arcas Tomeo, Lucía Llanos-Jiménez, Óscar González-Lorenzo, Andrea Kallmeyer, Borja Ibáñez, María Luisa González-Casaus, Gonzalo Hernández, Cristina Espadas, Óscar Lorenzo, Ignacio Mahíllo-Fernández, Jesús Egido, Pedro Luis Sánchez, Francisco Marín, Fernando Alfonso

**Affiliations:** 1 Department of Cardiology, IIS-Fundación Jiménez Díaz, Avenida Reyes Católicos 2, 28040 Madrid, Spain; 2 Department of Medicine, Autónoma University, Madrid, Spain; 3 Vascular Research Laboratory, IIS-Fundación Jiménez Díaz, Madrid, Spain; 4 CIBERCV, Instituto de Salud Carlos III (CNIC), Madrid, Spain; 5 Centro Nacional de Investigaciones Cardiovasculares Carlos III (CNIC), Madrid, Spain; 6 Department of Cardiology, University Hospital La Princesa, Madrid, Spain; 7 Department of Cardiology, University Hospital Guadalajara, Guadalajara, Spain; 8 Department of Cardiology, Hospital Clínico, Universitario Virgen de la Arrixaca, CIBERCV, IMIB-Arrixaca, Murcia, Spain; 9 Department of Cardiology, University Hospital of Salamanca-IBSAL, University of Salamanca, Salamanca, Spain; 10 Department of Cardiology, University Hospital Puerta de Hierro, Madrid, Spain; 11 Department of Radiology, Fundación Jiménez Díaz, Madrid, Spain; 12 Research Unit, IIS-Fundación Jiménez Díaz, Madrid, Spain; 13 Department of Laboratory Medicine, La Paz University Hospital, Madrid, Spain; 14 FAES FARMA, Leioa, Vizcaya Spain; 15 CIBERDEM, Madrid, Spain; 16 Department of Nephrology, Fundación Jiménez Díaz, Madrid, Spain; 17 Present Address: ESAME Biomedical, Getxo, Vizcaya Spain

**Keywords:** Acute myocardial infarction, Calcifediol, Cardiac magnetic resonance imaging, Remodelling, Vitamin D, Cardiology, Diseases, Medical research

## Abstract

No clinical trial has yet assessed the effect of vitamin D on patients experiencing ST-segment elevation myocardial infarction (STEMI). In this multicentre, randomised (2:1), double-blind, placebo-controlled trial, 93 patients with anterior STEMI were randomised to calcifediol or placebo, for 12 months. The primary endpoint was left ventricular remodelling, defined as a 15% increase in left ventricular end-diastolic volume (EDV) on magnetic resonance imaging. Sixty-five patients were allocated to calcifediol and 28 to placebo. Calcidiol levels increased in the intervention group (20.0[8.4, 36.5] vs. 2.0[− 0.3, 4.5] ng/mL, *p* < 0.001). No differences were observed in the incidence of left ventricular remodelling (26.2% vs. 21.4%; *p* = 0.824) and in these secondary end points: increment in end-diastolic volume (6.0[− 6.5, 21.6] vs. 6.1[− 8.1, 23.8] mL; *p* = 0.773), end-systolic volume (− 6.9[− 15.9, 4.5] vs. 1.3[− 20.9, 17.2] mL; *p* = 0.908), ejection fraction (5.1[2.1, 10.0] vs. 7.7[− 0.9, 11.0]%; *p* = 0.992), and in plasma levels of N-terminal pro-brain natriuretic peptide, galectin-3, ST2, growth differentiation factor-15, fibroblast growth factor-23 (FGF23), high-sensitivity C-reactive protein, and monocyte chemoattractant protein-1. Similar results were observed in patients with baseline FGF23 above the median, in those with calcidiol < 30 ng/mL, and in the subgroup with ejection fraction ≤ 40%. In conclusion, calcifediol does not improve left ventricular remodelling or affect biomarkers related to heart failure and inflammation following a STEMI.

**Trial registration**: NCT02548364. https://clinicaltrials.gov/study/NCT02548364?term=VITDAMI%20Vitamin%20D%20in%20acute%20myocardial%20infarction&rank=1.

**Supplementary Information:**

The online version contains supplementary material available at 10.1038/s41598-026-45330-6.

## Introduction

Vitamin D (VD), fibroblast growth factor-23 (FGF-23), parathyroid hormone (PTH), phosphate and klotho levels, are the main components of the human mineral metabolism system. Although this system was traditionally known to be dysregulated in patients with chronic kidney disease, patients with normal estimated glomerular filtration rate may have low plasma levels of calcidiol (25OH Vitamin D) and high plasma levels of FGF23, PTH and phosphate. Notably, all these abnormalities have been associated in different studies with cardiovascular damage^1–13^.

Low plasma VD levels play an important role in the state of the myocardium. Administration of calcitriol improves fibrosis in nephrectomized rats^14^, and the VD analogue paricalcitol inhibits myocardial fibrosis in a model of left ventricular (LV) pressure overload in mice^15^. In spite of this, in humans, VD supplements administered for one year failed to improve consistently LV remodelling in patients with chronic renal failure^16^ and in patients with long-term LV systolic dysfunction^17^. These negative results could have been due to the chronic nature of the studied disorders. Against this background, we hypothesized that a potential anti-remodelling effect of VD supplements could be more evident in patients with acute anterior ST-segment elevation myocardial infarction (STEMI), as a LV remodelling process takes place following this event^18^.

The VITDAMI (VITamin D in Acute Myocardial Infarction) is a prospective, multicentre, randomized, double-blind, placebo-controlled trial in patients with anterior STEMI designed to test the effect of calcifediol on LV remodelling and in the changes of on plasma levels of biomarkers related to heart failure and inflammation.

## Methods

### Trial population

Patients 40–85 years of age admitted to the 5 Spanish University hospitals (Fundación Jiménez Díaz, La Princesa, Puerta de Hierro [Madrid] La Arrixaca [Murcia] and University Hospital of Salamanca [Salamanca]) for an anterior STEMI undergoing primary angioplasty and who agreed to participate and signed the informed consent, were eligible for randomization. Inclusion and exclusion criteria have been reported previously^19^ (Table A, Supplemental material). Initially, we set a lower limit of 30% ejection fraction (EF) at the first echocardiogram to avoid recruiting patients candidates for an automatic implantable cardioverter-defibrillator who could not undergo the final magnetic resonance imaging (MRI). However, as many patients showed a significant EF recovery at the first MRI, in April 2016 the steering committee decided to lower this threshold to 25%.

### Ethic aspects

This trial was approved by the regional Ethical Committee of the Community of Madrid and by the Competent National Health Authority (Spanish Agency of Medicines and Medical Products [AEMPS]). All research was performed in accordance with relevant guidelines and regulations set out in the Declaration of Helsinki. The investigators took out liability insurance to cover the patients against potential adverse consequences of their participation in the trial. All patients signed an informed consent before participating. A specific section to consent for the storage of biological samples was included in the informed consent form. Clinical trial pharmacovigilance and monitoring were performed by SCReN (academic Spanish Clinical Research Network). The trial is registered in ClinicalTrials.gov (NCT02548364) and in the EU Clinical Trials Register (EudraCT 2014-004512-11).

### Trial design

The VITDAMI is a prospective, randomized, multicentre, double-blind, placebo-controlled trial. One-hundred and ten patients with anterior STEMI were included after successful primary angioplasty. After signing the informed consent, they underwent a cardiac MRI and blood extraction for plasma storage and were then randomly assigned (2:1) to receive on a double-blind basis an oral dose of 0.266 mg calcifediol (equivalent to 15,960 units) (soft gelatine [Hidroferol® SGC]) or placebo every 15 days (± 1 day), for one year. Randomization was computer-generated by the statistician at the coordinating centre and it was block-balanced. Patients were medically managed according to the European Society of Cardiology clinical practice guidelines for STEMI^20^.

At 1.5 months, calcidiol and plasma levels were assessed and the dose of the study medication was modified by telephone contacts according to the following protocol: if calcidiol was > 70 ng/mL or calcium > 10.5 mg/dl, the study medication was stopped; if calcidiol was 30–70 ng/mL with calcium ≤ 10.5 mg/dl, the dose was reduced to one capsule every month; if calcidiol was < 30 ng/mL and calcium ≤ 10.5 mg/dl, the dose was not changed. At months 3 and 9, adverse effects were checked by telephone. At month 6, patients were seen by the investigators in outpatient clinics to rule out adverse effects and to collect empty blisters to confirm compliance with the study medication. At month 12, cardiac MRI and biomarker assessment were performed, and the study medication was discontinued. One month after the last dose, a follow-up clinical visit was performed by telephone to record any possible adverse events. Patient recruitment started in November 2015 and ended in January 2023.

### Cardiac magnetic resonance imaging

Cardiac MRI was carried out to study LV end-diastolic (EDV) and end-systolic (ESV) volumes, LV mass, myocardial oedema and fibrosis/necrosis, and microvascular obstruction, as a parameter related to cardiac remodelling^21^. Scans were performed with 1.5 Tesla devices at each participating centre. All MRI studies were analysed by two investigators blinded to treatment allocation at a central corelab in the coordinating center, Fundación Jiménez Díaz, using the QMass system (7.6). Inter- and intraobserver variability was assessed in 15% of the MRI studies. A detailed description of the cardiac MRI procedures is given in the Supplemental Material.

### Blood extraction and assessments

At blood withdrawal, 26 mL was taken, of which 18 mL was placed in EDTA tubes and 8 mL in tubes without EDTA. Blood underwent centrifugation for 10 min at 2500 G and plasma was stored at − 80 °C. The study of mineral metabolism was performed at the clinical biochemistry laboratory of the La Paz university hospital and the remaining determinations were carried out at the biochemistry and vascular pathology laboratories at Fundación Jiménez Díaz. The investigators performing blood assessments were blinded to treatment allocation. Calcidiol levels were quantified using chemiluminescent immunoassay (CLIA) with LIAISON ®XLanalyzer (total VD Assay DiaSorin, Saluggia, Italy). FGF-23 was assessed by an ELISA that recognizes epitopes in the carboxyl-terminal portion of FGF-23 (Human FGF-23, C-Term, Immutopics Inc, San Clemente, CA), klotho was studied by ELISA (soluble alpha-klotho ELISA, IBL International, Hamburg, Germany), intact PTH by a second-generation automated chemiluminescent method (2010 platform Elecsys, Roche Diagnostics, Mannheim, Germany), and phosphate was assessed by an enzymatic method (Integra 400 analyzer, Roche Diagnostics, Mannheim, Germany). N-terminal probrain natriuretic peptide (NT-proBNP) levels were determined by immunoassay (VITROS, Orthoclinical Diagnostics, USA), high-sensitivity C-reactive protein by immunoturbidimetric latex and determinations of lipids, glucose and creatinine were carried out by standard methods (ADVIA 2400 Chemistry System, Siemens, Germany). Plasma levels of monocyte chemoattractant protein-1 (MCP-1) and galectin-3 were determined in duplicate using commercially available ELISA kits (BMS279/2, Bender MedSystems, Burlingame, California; and DCP00, R&D Systems, Minneapolis, Minnesota). Growth Differentiation factor-15 (GDF15) and ST2 levels were determined by immunoluminiscent immunoassay with the automated ELLA system (Protein Simple custom assays ref.: SPCK-PS-000269 [GDF15] and SPCKB-PS-000221 [sST2], Bio-techne, Minneapolis, MN, USA). Albumin and calcium were assessed by standard methods (ADVIA 2400 Chemistry System, Siemens, Germany).

### Trial endpoints

The primary endpoint was the proportion of patients that developed LV remodelling, predefined by protocol as an increase of≥ 15% in the EDV measured by cardiac MRI at the end of the study as compared with the baseline assessment. We used this cut-off given that in previous studies this value was above the intra- and interobserver variability^22^.

The main secondary endpoints were the percentage change in LV EDV, ESV, and EF. Other secondary endpoints were the change in stroke volume, LV mass, size of the necrotic/fibrotic area, myocardial oedema, and change in plasma levels of NT-proBNP, galectin-3, ST2, GDF15, calcidiol, FGF23, PTH, phosphate, klotho, galectin-3, high-sensitivity C-reactive protein, and MCP-1.

The safety endpoints were the rate of adverse events including death, heart failure, or any acute coronary syndrome.

### Statistical analysis

As there are no published data on the effect of VD on LV remodelling in patients with STEMI, a formal sample size calculation could not be performed. Accordingly, the trial may be considered as exploratory for the primary endpoint.

Analyses of treatment effect were performed per protocol, as prespecified^19^. Quantitative variables were described as median (interquartile range). Differences from baseline and changes in the variables assessed between the calcifediol and the placebo groups were studied using the Student’s t-test for quantitative variables with normal distribution, and with the Mann–Whitney test for those not following a normal distribution. Qualitative variables were described as percentages and compared using a chi-square test.

Interobserver variability was assessed by the estimation of intraclass correlation coefficients. Statistical tests were considered significant when “*p*” (two-tailed) was < 0.05. Analyses were performed with SPSS 19.0 statistical package.

## Results

**Fig. 1:**
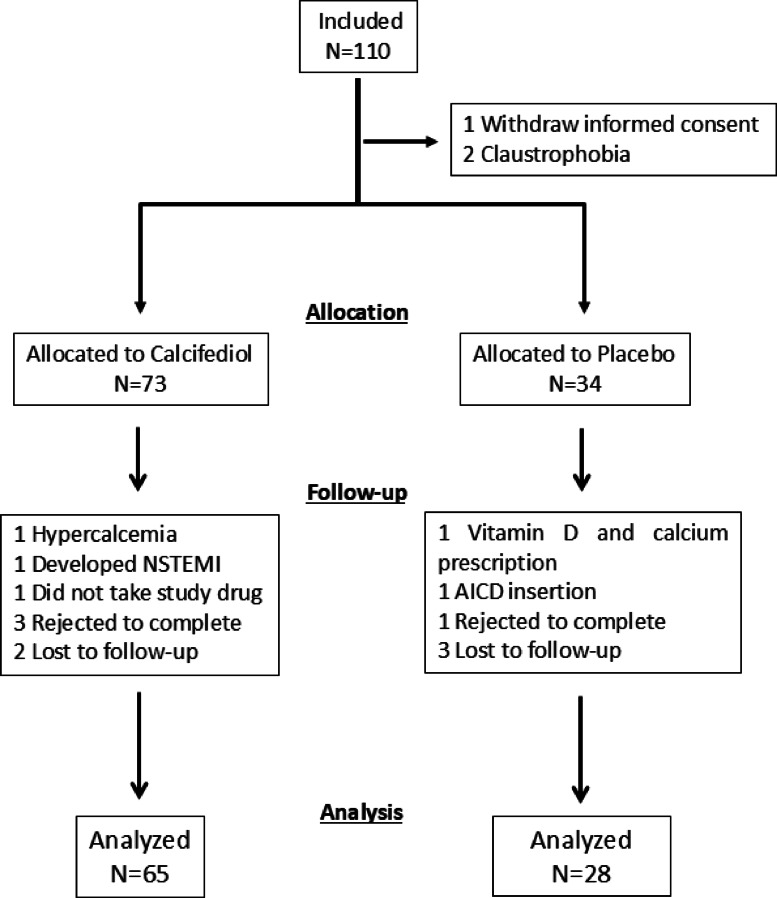
CONSORT diagram. AICD: Automatic implantable cardioverter-defibrillator; NSTEMI: Non-ST-elevation myocardial infarction.

Three patients did not enter the trial (1 withdraw the informed consent, and 2 had claustrophobia). From the remaining 107 patients 14 did not complete the trial: 1 developed hypercalcemia, 1 had a non-STEMI, 1 did not take the study drug, 1 received a prescription of vitamin D and calcium, 1 received an automatic implantable cardioverter-defibrillator, 4 rejected to complete, and 5 were lost to follow-up (Fig. 1). Therefore, 93 patients were available for the main analysis; 65 randomized to calcifediol and 28 to placebo. There were no differences in age, gender or prevalence of cardiovascular risk factors between the two groups (Table 1). At baseline, the calcifediol group and placebo groups had similar EDV, ESV, EF, stroke volume, and LV edema, while late gadolinium enhancement was larger in the placebo group. The MRI findings remained similar after adjusting for body surface area (Table 1). Similarly, plasma levels of NT-proBNP, galectin-3, ST2, GDF15, high-sensitivity C-reactive protein, MCP-1, calcidiol, FGF23, PTH, phosphate, klotho, and medical therapy prescribed at discharge, were not different between the two groups (Table 1).

**Table 1:** Baseline data in calcifediol and placebo groups.

Variable	Placebo (*N* = 28)	Calcifediol (*N* = 65)	*P*
**Clinical data**			
Age (years)	54.0 (51.0, 59.5)	58.0 (50.0, 67.0)	0.097
Gender (male, %)	92.9	86.2	0.495
Race (Caucasian, %)	96.4	95.4	0.768
Body Surface area (m^2^)	1.91 (1.81, 2.01)	1.94 (1.83, 2.09)	0.715
Body mass index (Kg/m^2^)	26.8 (24.9, 30.3)	26.8 (24.6, 29.9)	0.989
Diabetes (%)	10.7	16.9	0.541
Hypertension (%)	39.3	41.5	1.000
Smoker (%)	60.7	52.3	0.603
Dyslipidemia (%)	57.1	36.9	0.114
Culprit lesion			0.596
Proximal LAD (%)	53.6	43.1	
Middle LAD (%)	42.9	53.8	
Distal LAD (%)	3.6	1.5	
First diagonal (%)	0.0	1.5	
Number of vessels (%)			0.220
1	89.3	75.4	
2	10.7	15.4	
3	0.0	9.2	
Number of vessels treated	1.0 (1.0, 1.0)	1.0 (1.0, 1.0)	0.189
Complete revascularization (%)	92.9	87.7	0.718
**Analytical data**			
Estimated glomerular filtration rate (mL/min/1.73 m^2^)	83.1 (72.1, 93.8)	76.4 (63.6, 90.5)	0.214
Total cholesterol (mg/dl)	176 (153, 199)	184 (167, 205)	0.976
Low-density lipoprotein (mg/dl)	118 (92, 139)	117 (103, 137)	0.949
High density lipoprotein (mg/dl)	36.5 (29.8, 47.5)	39.0 (35.0, 45.0)	0.412
Triglycerides (mg/dl)	143 (74.8, 189)	122 (84.0, 158)	0.180
Hemoglobin (g/dl)	14.6 (14.0, 15.8)	15.2 (14.1, 16.1)	0.653
Glycemia (mg/dl)	128 (112, 160)	134 (116, 160)	0.258
Calcidiol (ng/mL)	25.0 (19.0, 34.5)	25.0 (16.2, 33.0)	0.834
Calcitriol (pg/mL)	44.0 (32.2, 70.0)	40.0 (30.0, 52.0)	0.065
FGF23 (RU/mL)	80.5 (63.2, 99.0)	79.0 (54.2, 101)	0.831
Klotho (pg/mL)	602 (514, 738)	582 (496, 734)	0.295
Parathormone (ng/mL)	42.7 (33.3, 44.6)	45.7 (33.6, 59.6)	0.329
Phosphate (mg/dl)	4.40 (3.90, 4.85)	4.10 (3.60, 4.70)	0.248
Calcium (mg/dL)	9.7 (9.40, 10.0)	9.6 (9.20, 10.0)	0.131
Hs-CRP (mg/L)	23.3 (10.2, 52.7)	16.7 (7.9, 32.1)	0.185
NT-proBNP (ng/L)	1210 (872, 2555)	1180 (632, 2335)	0.481
Galectin-3 (ng/mL)	10,204 (8116, 12,795)	9196 (7190, 11,291)	0.087
MCP1 (pg/mL)	107.0 (97.3, 142.0)	129.0 (102.0, 150.0)	0.250
ST2 (pg/mL)	20,100 (15,545, 26,898)	18,762 (15,360, 24,487)	0.567
GDF15 (pg/mL)	877 (638, 1112)	912 (688, 1364)	0.578
**Medication at discharge**			
Acetylsalicylic acid (%)	96.4	95.4	1.000
P2Y12 inhibitor (%)	96.4	100	0.301
Statin (%)	100.0	95.4	0.551
Diuretic (%)	32.1	18.5	0.239
ARNI (%)	3.6	0.0	0.301
Betablocker (%)	85.7	86.2	1.000
ACEI / ARB (%)	89.3	90.8	1.000
MRA (%)	35.7	41.5	0.650
SGLT_2_ inhibitor (%)	7.1	6.2	1.000
Number of anti-remodelling drugs (%)			0.804
0	3.6	3.1	
1	14.3	7.7	
2	42.9	52.3	
3	35.7	35.4	
4	3.6	1.5	
**Magnetic Resonance Imaging**			
End-diastolic volume (mL)	174 (134, 196)	150 (119, 186)	0.114
End-diastolic volume by BSA (mL/m^2^)	87.7 (73.0, 100)	78.9 (65.7, 89.6)	0.054
End-systolic volume (mL)	95.0 (73.0, 111.0)	77.4 (58.9, 91.2)	0.096
End-systolic volume by BSA (mL/m^2^)	46.7 (35.6, 56.3)	39.8 (31.7, 48.2)	0.056
Ejection fraction (%)	45.2 (40.3, 51.2)	48.5 (40.2, 55.6)	0.182
LV mass (g)	119 (104, 132)	121 (97, 134)	0.929
LV mass by BSA (g/m^2^)	62.5 (52.6, 70.1)	60.8 (53.1, 67.2)	0.986
Stroke volume (mL)	73.0 (58.7, 88.1)	72.9 (62.7, 79.4)	0.580
Stroke volume by BSA (mL/m^2^)	37.4 (32.1, 43.0)	37.5 (30.7, 40.9)	0.493
LV edema (mL)	31.1 (6.8, 44.2)	28.4 (16.0, 42.5)	0.518
LV edema by BSA (mL/m^2^)	14.9 (3.50, 23.8)	15.0 (8.28, 21.5)	0.560
Gadollinium enhancement (g)	32.3 (22.1, 38.6)	23.7 (14.2, 33.6)	**0.047**
Gadollinium enhancement by BSA (g/m^2^)	16.4 (11.4, 20.2)	12.8 (7.77, 17.4)	**0.048**

*ACEI* angiotensin-converting enzyme inhibitor, *ARB* angiotensin receptor blocker, *ARNI* angiotensin receptor-neprilysin inhibitor, *BSA* body surface area, *estimated glomerular filtration rate*: estimated glomerular filtration rate, *FGF23* fibroblast growth factor-23, *GDF15* Growth Differentiation Factor-15, *Hs-CRP* high-sensitivity C-reactive protein, *LAD* left anterior descending coronary artery, *LV* left ventricle, *MCP-1* monocyte chemoattractant protein-1, *MRA* mineralcorticoid receptor antagonist, *NT-proBNP* N-terminal pro-brain natriuretic peptide. Significant *P *values are in [bold].

The adherence to the study medication was high, with 10.8% patients in the VD group and 17.9% in the placebo group having forgotten at least one dose (*p* = 0.501). In the VD group, two patients (3.1%) missed two doses and five patients (7.7%) forgot a single dose, while in the placebo group one patient (3.6%) forgot four doses and 4 patients (14.3%) missed only one.

**Fig. 2:**
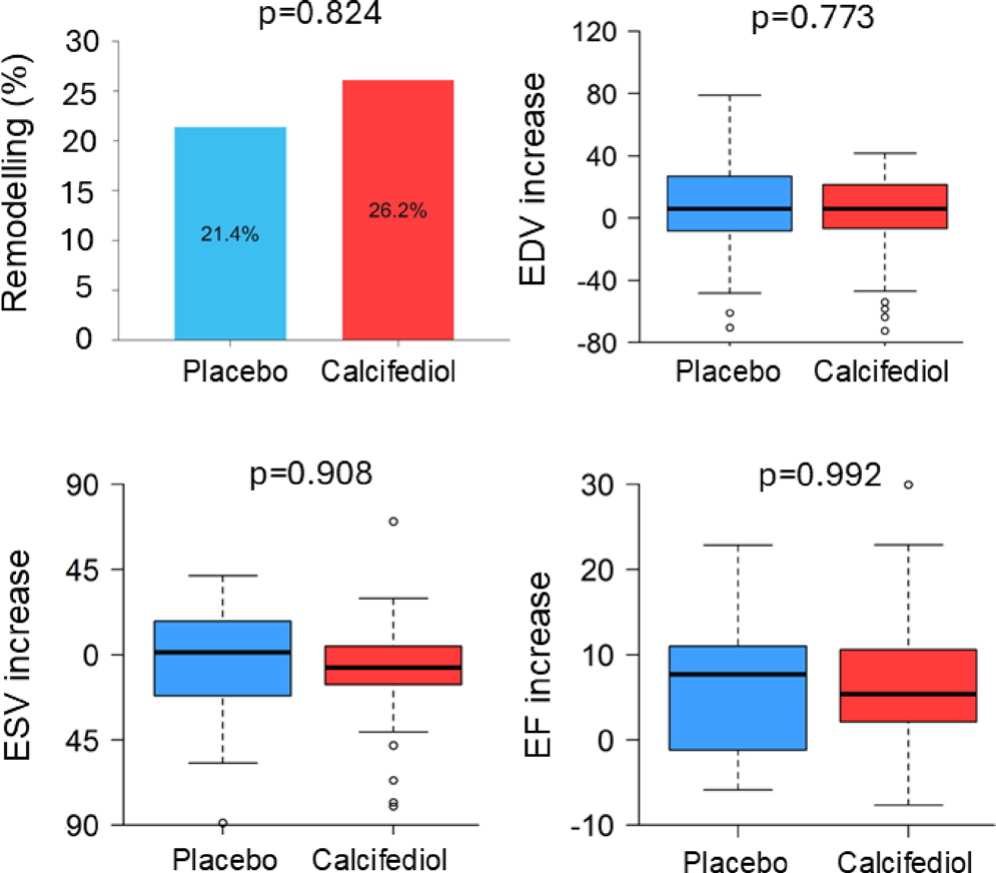
Comparison of primary and secondary endpoints in calcifediol versus placebo group. There were no significant differences between the percentage of patients developing remodelling nor in the changes in end-diastolic volume (EDV), end-systolic volume (ESV) or left ventricular ejection fraction (EF) after one year of treatment with calcifediol as compared with placebo. Box plots show the median and interquartile range, with whiskers extending up to 1.5 times the interquartile range; points beyond the whiskers show the outliers.

**Fig. 3:**
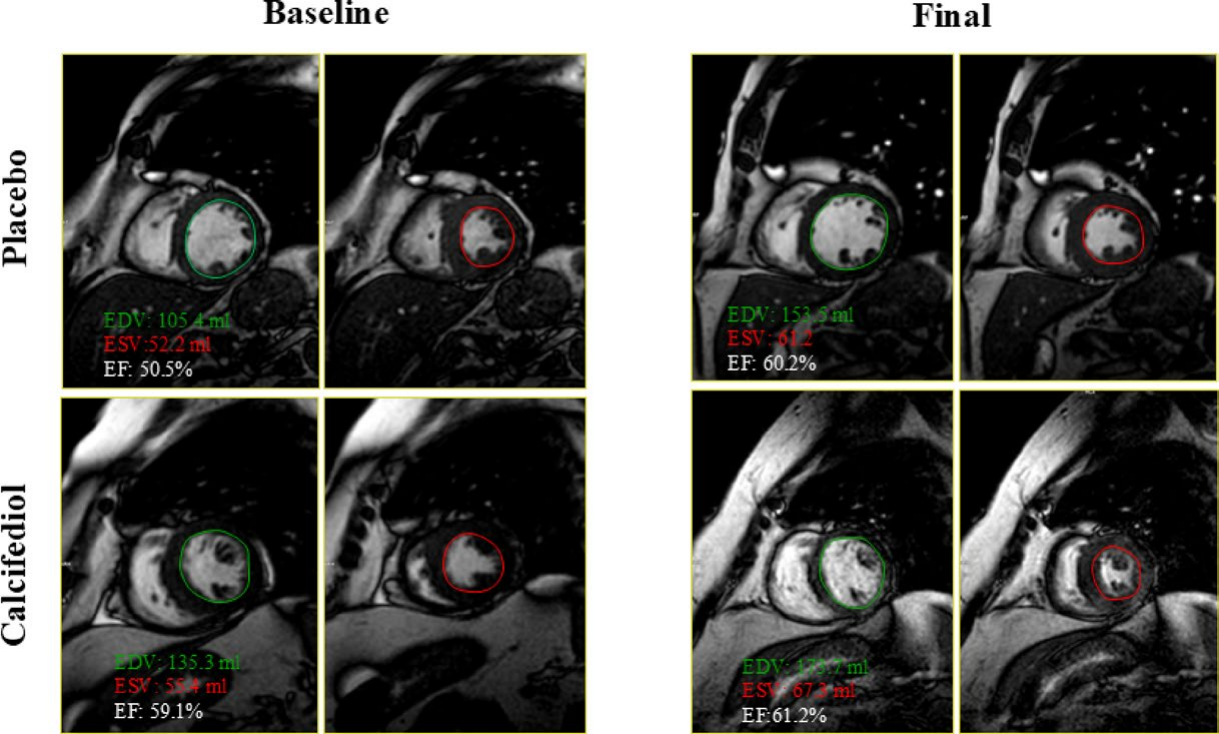
Representative examples of baseline and final cardiac magnetic resonance imaging (MRI) in a patient on placebo (number 33) and another treated with calcifediol (number 19). Green lines mark the left ventricular end-diastolic volume (EDV) and red lines correspond to the end-systolic volume (ESV). EF: Ejection fraction.

The percentage of patients who developed LV remodelling was 26.2% in the calcifediol group and 21.4% in the placebo group (*p* = 0.824) (Fig. 2). The increase in EDV and EF, and the decrease in ESV were not significantly different between the two groups (Figs. 2 and 3, and Table 2). Similarly, other parameters assessed -LV mass, stroke volume, LV edema, and the extent of the region showing late gadolinium enhancement- did not differ in the final vs the initial MRI (Table 2). When absolute MRI measurements were corrected for body surface area the results did not change (Table 2).

**Table 2:** Changes in left ventricular measurements assessed by magnetic resonance imaging.

Variable	Placebo	Calcifediol	*P*
End-diastolic volume (mL)	6.1 (− 8.1, 23.8)	6.0 (− 6.5, 21.6)	0.773
End-diastolic volume/BSA (mL/m^2^)	3.6 (− 4.5, 11.3)	3.0 (− 3.4, 10.8)	0.853
Percent increase in end-diastolic volume	4.1 (− 5.9, 12.9)	3.99 (− 3.4, 15.3)	0.730
End-systolic volume (mL)	1.3 (− 20.9, 17.2)	− 6.9 (− 15.9, 4.5)	0.908
End-systolic volume/BSA (mL/m^2^)	0.5 (− 10.1, 9.3)	− 3.6 (− 7.9, 2.4)	0.959
LV ejection fraction (%)	7.7 (− 0.9, 11.0)	5.1 (2.1, 10.0)	0.992
LV mass (g)	− 13.4 (− 30.3, − 5.8)	− 13.9 (− 28.1, − 4.6)	0.629
LV mass/BSA (g/m^2^)	− 7.1 (− 17.0, − 3.1)	− 7.8 (− 13.9, − 2.1)	0.655
Stroke volume (mL)	11.0 (− 2.1, 20.7)	11.4 (2.4, 17.9)	0.991
Stroke volume/BSA (mL/m^2^)	5.4 (− 1.2, 10.4)	5.7 (1.1, 9.7)	0.983
LV Edema (mL)	− 24.0 (− 43.7, − 3.8)	− 23.5 (− 36.9, − 7.6)	0.821
LV Edema/BSA (mL/m^2^)	− 11.7 (− 23.7, − 2.0)	− 13.3 (− 19.5, − 3.6)	0.168
Gadollinium enhancement (g)	− 11.8 (− 20.8, − 0.2)	− 8.4 (− 15.1, − 1.2)	0.552
Gadollinium enhancement/BSA (g/m^2^)	− 5.9 (− 11.2, − 0.1)	− 4.7 (− 8.0, − 0.7)	0.538

Changes are displayed as final-initial values. *BSA* body surface area, *LV* left ventricle.

The interobserver correlation (*r* = 0.907–0.999) and agreement (intraclass correlation coefficient = 0.922–0.999) were excellent (*p* < 0.001 for all) (Table B, Supplemental Appendix). Similar results were obtained for intraobserver correlation (*r* = 0.867 to 0.988) and agreement (intraclass correlation coefficient = 0.928–0.991) (*p* < 0.001 for all) (Table C, Supplemental Appendix).

**Fig. 4:**
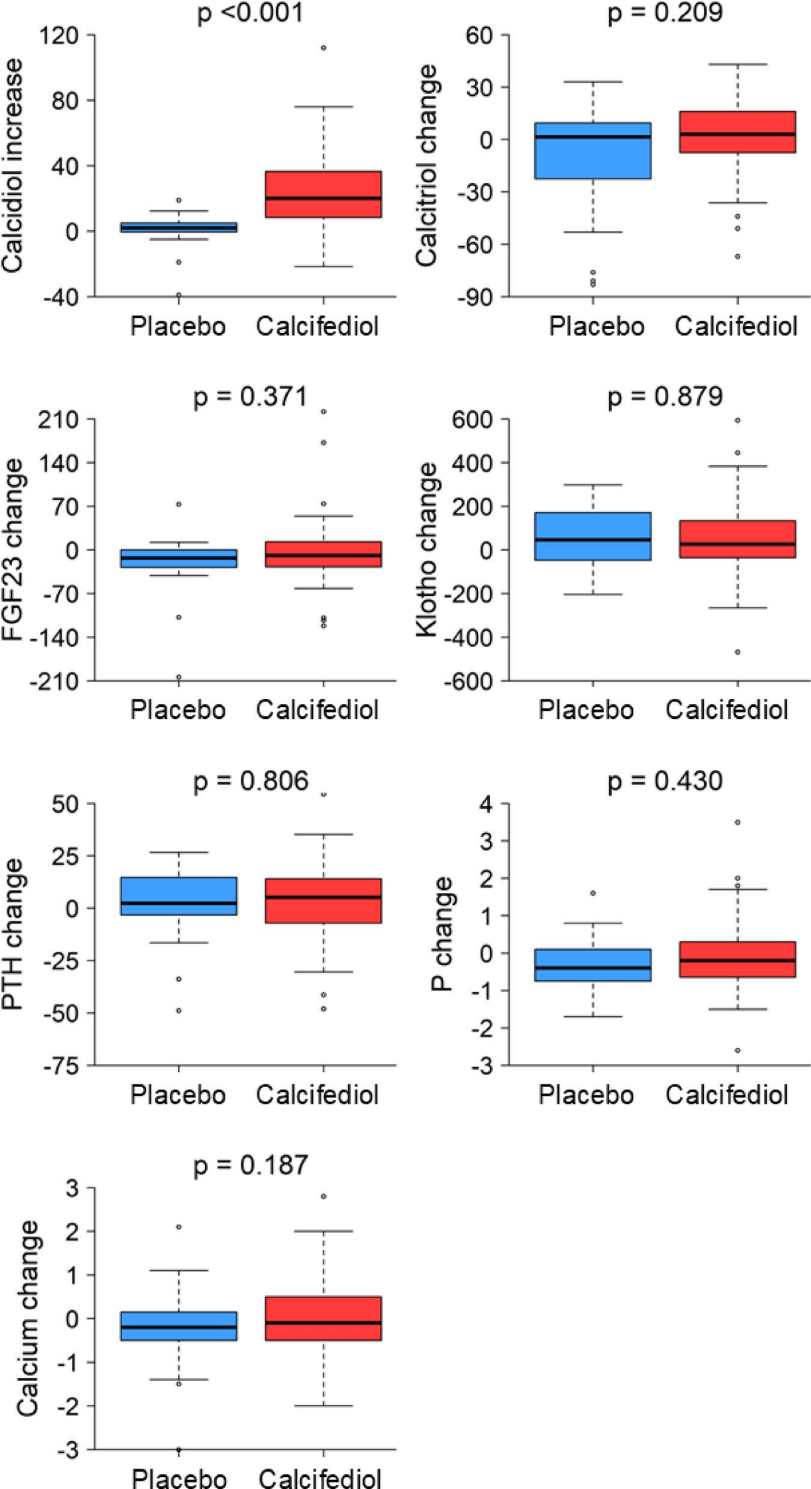
Changes in plasma concentrations of the components of mineral metabolism. In the active treatment group there is a significant increase in plasma calcidiol levels without changes in the concentrations of the other molecules assessed. FGF23: Fibroblast growth factor-23; P: Phosphate; PTH: Parathormone. Box plots show the median and interquartile range, with whiskers extending up to 1.5 times the interquartile range; points beyond the whiskers show the outliers.

Figure 4 shows that calcidiol levels increased in the calcifediol versus the placebo group (20.0[8.4, 36.5] vs. 2.0[− 0.3, 4.5] ng/mL; *p* < 0.001) while there were no significant changes in plasma levels of calcitriol (3.0[− 7.5, 16.0] vs. 1.5[− 22.2, 9.3] pg/mL; *p* = 0.209), FGF23 (− 9.0[− 27.0, 13.0] vs. − 13.0[− 27.5, 0.0] RU/mL; *p* = 0.371), klotho (26.0[− 36.0, 134.0] vs. 46.5 [− 47.0, 168.0] pg/mL; *p* = 0.879), PTH (5.2[− 7.2, 14.0] vs. 2.4[− 2.6, 13.6] ng/mL; *p* = 0.806), phosphate (− 0.20[− 0.65, 0.30] vs. − 0.40[− 0.75, 0.10] mg/dL; *p* = 0.430), or calcium (− 0.10[− 0.50, 0.50] vs. − 0.20[− 0.50, 0.13] mg/dL; *p* = 0.187). Sun exposure, as reported by patients, was lower in the calcifediol than in the placebo group (2.0[1.8, 4.0] vs. 4.0 [2.5, 6.0] hours per day, respectively; *p* = 0.010).

Similarly, patients treated with calcifediol showed no differences in the increase in NT-proBNP (− 1039[− 1937, − 520] vs. − 1024[− 2166, − 697] ng/L; *p* = 0.702), galectin-3 (− 2031[− 3920, − 290] vs. − 3332[− 4948, − 340] ng/mL; *p* = 0.260), ST2 (− 3271[− 10259, 462] vs. − 3231[− 6582, 1706] pg/mL; *p* = 0.202), GDF15 (149[− 80, 469] vs. 138[− 135, 433] pg/mL; *p* = 0.742), high-sensitivity C-reactive protein (− 13.6 [− 30.2, − 5.7] vs. − 14.1 [− 57.0, − 6.7] mg/L; *p* = 0.269), MCP-1 (32.7[− 2.3, 66.5] vs. 35.0[24.0, 78.0] pg/mL; *p* = 0.183), and estimated glomerular filtration rate (0.49[− 6.7, 11.2] vs. 7.0 [− 3.7, 17.8]; *p* = 0.261) as compared with those of the placebo group.

We then assessed in a prespecified sensitivity analysis the effect of colecalcifediol treatment in patients with high FGF23^19^. Given the lack of a consistently accepted normal range of plasma FGF-23 values we used the median (79.0 RU/mL; *N* = 44) as the cut-off point. Thirty-six patients received calcifediol and 8 placebo. There were no differences in baseline clinical data except for estimated glomerular filtration rate (65.7[59.6, 84.6] vs. 83.2[73.4, 94.8] mL/min/1.73 m^2^; *p* = 0.015), which was lower in the calcifediol than in the placebo group (Table D, Supplemental Appendix). Cardiac MRI parameters were also similar except for a smaller extension of gadolinium enhancement in the vitamin D group in absolute values and after correction for body surface area (Table D, Supplemental Appendix).

Patients who received calcifediol showed no differences in the incidence of LV remodelling as compared with the placebo group (30.0% vs. 21.4% respectively; *p* = 0.722). Similarly, there were no differences in the change in EDV (6.1(− 5.7, 20.5) vs. 7.7(− 24.0, 12.7) mL; *p* = 0.841), ESV (− 7.0[− 13.2, 11.3] vs. − 4.8 [− 28.1, 17.6] mL; *p* = 0.547), ejection fraction, LV mass, stroke volume, LV edema, or extension of gadolinium enhancement, between the baseline and the final visit as compared with those on placebo; the results did not change after correcting absolute values for body surface area (Table E, Supplemental Appendix). As expected, there was a significant increase in plasma calcidiol levels in patients who received calcifediol as compared with those on placebo (22.9[11.5, 33.0] vs. 3.0[0.0, 7.0] ng/mL, respectively; *p* < 0.001). Sun exposure was lower in the calcifediol than in the placebo group (2.0[1.0, 4.0] vs. 5.0[3.9, 8.6], hours per day respectively; *p* = 0.002). Changes in plasma levels of calcitriol, FGF23, klotho, PTH, phosphate, calcium, NT-proBNP, galectin-3, ST2, GDF15, high-sensitivity C-reactive protein, and MCP-1, and in estimated glomerular filtration rate were not different between the two groups (data not shown).

The effect of colecalcifediol was also assessed in the prespecified sensitivity analysis of patients with low or suboptimal calcidiol levels^23^ at baseline^19^. At baseline, 58 patients had plasma calcidiol levels lower than 30 ng/mL. Forty received calcifediol and 18 placebo. There were no differences between clinical, analytical and MRI parameters at baseline, except for a lower percentage of dyslipidemia in patients randomized to calcifediol (32.5% vs. 66.7%; *p* = 0.022) (Table F, Supplemental Appendix). There were no differences in the incidence of remodelling between the calcifediol and placebo groups (22.5% vs. 22.2% respectively; *p* = 1.000) in this subgroup of patients. Furthermore, there were no significant differences in the change in EDV (6.3[− 6.6, 19.3] vs. 6.1[− 2.5, 28.0] mL; *p* = 0.915), ESV (− 8.9[− 18.4, 4.7] vs. 6.8[− 16.0, 18.1] mL; *p* = 0.603), ejection fraction, or in any of the MRI parameters assessed both in absolute numbers and corrected for body surface area (Table G, Supplemental Appendix).

In this patient subset, there was also a significant increase in plasma calcidiol levels in the calcifediol group as compared with the placebo group (22.0[11.2, 35.8] vs. 3.0[0.5, 4.0] ng/mL, respectively; *p* < 0.001). Sun exposure was lower in the calcifediol than in the placebo group 2.0[1.0, 4.0] vs. 4.5[2.9, 8.1] hours per day respectively; *p* = 0.016). Changes in plasma levels of calcitriol, FGF23, klotho, PTH, phosphate, calcium, NT-proBNP, galectin-3, ST2, GDF15, high-sensitivity C-reactive protein, and MCP-1, and in estimated glomerular filtration rate were not different between the two groups (data not shown).

Finally, we conducted a sub-analysis in twenty-five patients with EF ≤ 40% at baseline. Seventeen received vitamin D and 8 placebo. There were no differences in baseline clinical data except for higher plasma galectin-3 levels in the placebo as compared with the calcifediol group (Table H, Supplemental Appendix). There were no differences in the incidence of remodelling between the calcifediol and the placebo groups (35.3% vs. 37.5%, respectively; *p* = 1.000). Furthermore, there were no significant differences in the change in EDV (− 1.35 [− 6.7, 22.4] vs. 8.7 [− 6.4, 40.6] mL; *p* = 0.511), ESV (− 11.3 [− 26.1, 4.5] vs. 4.0 [− 33.3, 26.4] mL; *p* = 0.440), ejection fraction, or in any of the MRI parameters assessed both in absolute numbers and corrected for body surface area (Table I, Supplemental Appendix).

 Among the prespecified adverse events, 1 patient in the calcifediol group developed a non-STEMI and was excluded as stated in the protocol. There were no deaths in any of the groups. Unstable angina, and heart failure were present in 1 patient (1.5%) in the calcifediol group and in none of the placebo group (*p* = 1.000 for both comparisons). Stable angina, coronary vasospasm, and atrial fibrillation were all present in 1 patient (1.5%) in the calcifediol group and in none of those who received placebo (*p* = 1.000 for both comparisons). Hypotension (1[1.5%] vs. 2[7.1%]; *p* = 0.215), and dyspnoea (2[3.1%] vs. 1[3.6%]; *p* = 1.000) did not have different incidences in the two groups. Similarly, no statistical differences were found between groups for the incidence of other adverse events (Table 3).

**Table 3:** Adverse events.

Variable	Placebo (*n* = 28)	Calcifediol (*n* = 65)	*P*
Unstable angina	0 (0.0%)	1 (1.5%)	1.000
Heart Failure	0 (0%)	1 (1.5%)	1.000
Stable angina	0 (0.0%)	1 (1.5%)	1.000
Coronary vasospasm	0 (0.0%)	1 (1.5%)	1.000
Atrial fibrillation	0 (0%)	1 (1.5%)	1.000
Hypotension	2 (7.1%)	1 (1.5%)	0.215
Dyspnea	1 (3.6%)	2 (3.1%)	1.000
Intraventricular thrombus	1 (3.6%)	0 (0%)	0.301
Non-cardiac chest pain	0 (0%)	6 (9.2%)	0.173
Abnormalities in hepatic analytics	2 (7.1%)	0 (0%)	0.088
Hyperglycemia	1 (3.6%)	1 (1.5%)	0.514
Musculoskeletal symptoms	0 (0%)	4 (6.2%)	0.312
Impairment in renal function	0 (0%)	1 (1.5%)	1.000
Gingival disorders	1 (3.6%)	1 (1.5%)	0.514
Diarrhea	1 (3.6%)	0 (0%)	0.301
Anxiety	2 (7.1%)	0 (0%)	0.088
Headache	0 (0%)	2 (3.1%)	1.000
Digestive bleeding	1 (3.6%)	2 (3.1%)	1.000
Tonsillitis	1 (3.6%)	0 (0%)	0.301
Hyperthyroidism	0 (0%)	2 (3.1%)	1.000
Gout	0 (0%)	1 (1.5%)	1.000
Epistaxis	0 (0%)	1 (1.5%)	1.000
Sleep apnea	0 (0%)	2 (3.1%)	1.000

## Discussion

Vitamin D deficiency plays an important role in the development of myocardial disease, as it is associated with LV hypertrophy and systolic dysfunction, biatrial enlargement, metabolic changes, inflammation, fibrosis and apoptosis in rat^24^. Studies performed in vitro and in several animal models of LV pressure overload showed that VD supplements attenuate LV hypertrophy, reduce cardiac fibrosis and decrease the expression of collagen, fibronectin and transforming growth factor-β (TGF-β), and improve systolic and diastolic function^14,15,25,26^.

Despite the previously described negative association between VD deficiency and cardiovascular disease^5,12^, previous clinical trials failed to convincingly demonstrate a benefit of VD supplements in terms of LV remodelling. The Paricalcitol Capsule Benefits in Renal Failure–Induced Cardiac Morbidity (PRIMO) trial, showed no improvement in ventricular mass index or any other remodelling parameters by administering paricalcitol, a selective activator of VD receptors, to patients with chronic renal failure^16^. The VitamIN D treatIng patients with Chronic heArT failure (VINDICATE) trial failed to show an improvement in the 6-min walking distance in patients with chronic LV systolic dysfunction using 4000 IU [100 mg] of vitamin D3 supplementation daily vs. placebo for one year^17^. Although VD supplementation was associated with an increase in the LV EF and a decrease in LV end-diastolic and end-systolic diameters, these variables were assessed by echocardiography, a less accurate technique than MRI, and they were only the secondary endpoints of the trial. The only exception was a meta-analysis including patients with heart failure and LV EF ≤ 40%, in which those receiving vitamin D supplements exhibited a significantly greater decrease of LV end-diastolic diameter than the control group^27^. However, the included studies used echocardiography instead of MRI, and the improvement of remodelling was weak, and close to the range of inter-observer and intra-observer variability.

We speculated that the PRIMO and VINDICATE trials could have failed to demonstrate a consistent benefit for LV remodelling because they were performed in patients with long-term adverse LV remodelling. In this chronic setting, it may be difficult to obtain a significant benefit with only one year of treatment. Given that STEMI triggers an evolving process of adverse remodelling^18^, we compared the effect of calcifediol vs placebo in this acute clinical setting. We specifically chose patients with anterior STEMI given that in this location the resulting LV function is usually worse than in other myocardial regions^28^. Despite using this selective approach, we failed to demonstrate a decrease in the incidence of cardiac remodelling by MRI (the study primary endpoint). Similarly, there were no differences in the development of LV EDV, ESV, EF, LV mass, areas of necrosis or oedema, in plasma levels biomarkers related to heart failure and inflammation, as well as in FGF23, PTH, klotho, and phosphate.

The observed lack of effect could have theoretically been related to the inability to increase calcitriol levels, given that this is the active form of VD. In this regard, in the VINDICATE study, a compound of calcitriol was used increasing both calcidiol and calcitriol plasma levels, leading to a decrease in PTH levels^17^, that was not observed in our study, where we only observed an increase of calcidiol levels. We cannot, therefore, rule out the possibility that using a compound containing calcitriol could have improved the results.

We have previously reported that the adverse predictive value of low plasma calcidiol levels in patients with coronary artery disease is more evident when combined with high plasma FGF-23 levels^5^. Similarly, other investigators have found that this combination is an excellent predictor of renal function deterioration in pre-dialysis patients^29^. For this reason, we included a prespecified a subgroup analysis for patients with FGF-23 levels higher than the median. However, although the sample size was small, in this subgroup the administration of calcifediol was also not associated with any beneficial effect on remodelling. Similar results were obtained when we analysed the effect of VD supplements in the subgroup of patients with low plasma calcidiol levels. As a whole, the results of these trials concur with those of the VITamin D and OmegA-3 TriaL (VITAL), where no cardiovascular effect of VD supplements was demonstrated in primary prevention^30^.

The main limitation of this trial was the small sample size. Unfortunately, a formal sample size calculation could not be performed as no previous trials of VD in patients with STEMI have been conducted. Data from the meta-analysis from Zhao et al.^27^ were not useful for this estimation given that they found a higher decrease in LV size in a population with LV dysfunction and remodelling at baseline. Instead of this, we were trying to demonstrate an effect of VD in preventing a future remodelling, that is, a smaller increase of LV volume, in STEMI patients before a potential remodelling begins. This limitation could have masked a potential effect of calcifediol. However, around 25% of patients developed remodelling and the trend did not favour the calcifediol group, suggesting that a larger sample size would not have been able to show a benefit of VD supplements. Furthermore, the use of cardiac MRI, the gold-standard technique for cardiac function assessment, limits the possibility of missing any benefit of calcifediol on LV remodelling. In addition, the absence of effect on all the biomarkers related to LV function and inflammation reinforces the results obtained by MRI. Also, the EF at baseline was higher than expected. Nevertheless, an exploratory sub-analysis for the subgroup of patients with an EF ≤ 40% at baseline was conducted, and no effect of VD supplements was found. The low number of women and non-caucasian participants did not allow studying the effects on these important subgroups of patients. Finally, we cannot exclude the possibility that the use of a different VD compound could have resulted in a beneficial effect.

In conclusion, the administration of calcifediol to patients with anterior STEMI treated with primary angioplasty does not prevent adverse LV remodelling at one year, and does not affect biomarkers related to heart failure and inflammation. These results are consistent in the predefined subgroups of patients with high plasma FGF23 or low plasma calcidiol levels at baseline, and also in the subgroup of patients with EF equal to or less than 40%.

## Electronic Supplementary Material

Below is the link to the electronic supplementary material.

Supplementary Material 1

## Data Availability

The data that support the findings of this study are available from the corresponding author upon reasonable request.

## Declarations

### Declaration of use of Artificial Intelligence

Artificial Intelligence has been used exclusively to improve the edition of small parts of this manuscript (DeepL) and to translate the Protocol file from Spanish to English.
