# Supplementary material for: Vitamin D in the prevention of left ventricular remodelling after an acute myocardial infarction: a randomized clinical trial
Source: Sci Rep. 2026 Apr 6;16:16486. doi: 10.1038/s41598-026-45330-6 (PMC13216248; doi:10.1038/s41598-026-45330-6)
Supplement: Supplementary file 1 — Supplementary Material 1 [file 41598_2026_45330_MOESM1_ESM.docx]

**SUPPLEMENTAL APPENDIX**

**INDEX**

Trial procedures………………………………………………….……..……………..…2

Table A. Exclusion Criteria………………………………………………….……..……3

Table B. Interobserver correlation and agreement………………………….…….……..4

Table C. Intraobserver correlation and agreement………………………………..……..4

Table D. Baseline data in calcifediol and placebo groups in patients with FGF23 higher than the median at baseline……………………………………………………………….5

Table E. Changes in Magnetic Resonance Imaging data in patients with FGF23 levels higher than the median at baseline………………………….……………………………7

Table F. Baseline data in calcifediol and placebo groups in patients with calcidiol lower than 30 ng/ml at baseline…………………………………………………………………8

Table G. Changes in Magnetic Resonance Imaging data in patients with calcidiol levels < 30 ng/ml………………………………………………………………………………….10

**Trial procedures**

Cardiac MRI was carried out to study cardiac function, LV mass, myocardial edema and myocardial fibrosis/necrosis. Scans were performed with 1.5 Tesla equipments at each participating centre. For assessment of global and segmental left ventricular function and LV mass, balanced steady-state free precession with sensitivity encoding fast parallel imaging technique (True FISP, SSFP) sequence was used for cine imaging. Images were obtained at short axis (8 mm slices from the apex to the base of the left ventricle), 4-chamber, 2-chamber, and 3-chamber. Myocardial edema was assessed matching the slice position of the cine images, using a T2-weighted triple IR turbo spin echo (STIR) sequence. For study of fibrosis/myocardial necrosis, gadolinium was intravenously administered. Delayed enhancement imaging to assess necrotic myocardium will be performed using IR Turbo-FLASH (3D-IR-GRE, 2D-IR-GRE, and PSIR sequences).

The necrotic/fibrotic area was defined as the area with delayed gadolinium up-take (high-intensity STIR signals) and myocardium at risk as the area with edema. Myocardium at risk and the necrotic size were expressed in grams. All the results were given in absolute numbers and normalized by Body Surface Area. Left ventricular end-diastolic and end-systolic volumes, the ejection fraction, and microvascular obstruction, a parameter related to cardiac remodeling(1) were determined. MRI studies were analyzed by investigators blinded to treatment allocation at a central core laboratory located at Fundación Jiménez Díaz with the QMass system (7.6). Fifteen percent of the MRI studies were randomly chosen to perform inter- and intraobserver variability.

**REFERENCES**

1. Romero J, Lupercio F, Díaz JC, Goodman-Meza D, Haramati LB, Levsky JM, et al. Microvascular obstruction detected by cardiac MRI after AMI for the prediction of LV remodeling and MACE: A meta-analysis of prospective trials. Int J Cardiol. 2016;202:344-8.

**Table A. Exclusion Criteria**

**_________________________________________________________________________**

-Death or complications compromising patient survival (i.e: cardiogenic shock) during the index event

-No primary angioplasty

-Impossibility to perform the desirable revascularization^1^

-Indication of cardiac transplant

-Surgical coronary revascularization during the index event

-Revascularization pending at the moment of discharge

- Complications that extend patient´s stay at the hospital for more than 7 days

-Absence of akinetic area in the anterior wall of the left ventricle

-Previous myocardial infarction

-Valve prosthesis

-Aortic stenosis with mean gradient>25 mm Hg (Doppler) and stenosis of any other valve

-Moderate to severe valve regurgitation

-Cardiomyopathy (Dilated, Hypertrophic or Restrictive)

-Severe left ventricle hypertrophy (wall thickness ≥17 mm in men and ≥16 mm in women)

-Conditions limiting survival

-Inflammatory or immune disease

-Estimated Glomerular Filtration Rate <40 ml/min/1.73 m^2^

-Intolerance to vitamin D supplements

-Indication or patient’s decision to take vitamin D or calcium

-Therapy with drugs that can interfere absorption, distribution, metabolism or excretion of Vitamin D:

- Anti-epileptic (Phenitoine, Phenobarbital, Primidone, other enzymatic inducers)
- Cholestiramine, cholestipol, Orlistat
- Penicillins
- Laxatives as paraphine and mineral oil
- Digitalis
- Magnesium salts
- Verapamil
- Steroids

-Disorders that can interfere the pharmacokinetics or pharmacodynamics of Vitamin D: Hepatic insufficiency, inflammatory bowel disease, sarchoidosis, Tuberculosis, or other granulomatous disorders.

-Plasma Calcium levels >10.5 mg/dl, history of hypercalciuria or calcium litiasis.

-Contraindications for Magnetic Resonance Imaging: claustrophoby, pacemaker, defibrillators, resynchronization devices, or high probability that they need them during the study (among them, patients with ejection fraction<25% at discharge)

-Impossibility to follow the patients

-Difficult compliance of the therapy as estimated by the investigator

-Patient rejects to participate and/or does not sign the informed consent

-Pregnancy or lactation period

-Patients participating in another clinical trial

^1^Severe stenoses (angiographic severity>70% or borderline with a positive intracoronary adenosine test) are allowed only in secondary vessels considered either not relevant by the investigator because of small vessel diameter, distal lesion, or technically unfeasible due to vessel calcification or tortuosity.

**Table B. Interobserver correlation and agreement**

| **VARIABLE** | **r** | **P** | **ICC** | **CI 95%** | **P** |
| --- | --- | --- | --- | --- | --- |
| End-diastolic volume (ml) | 0.933 | <0.001 | 0.999 | 0.997-1.000 | <0.001 |
| End-systolic volume (ml) | 0.983 | <0.001 | 0.991 | 0.971-0.997 | <0.001 |
| Ejection fraction (%) | 0.999 | <0.001 | 0.998 | 0.997-0.999 | <0.001 |
| LV mass (gr) | 0.983 | <0.001 | 0.991 | 0.970-0.997 | <0.001 |
| Stroke volume (ml) | 0.964 | <0.001 | 0.971 | 0.905-0.991 | <0.001 |
| LV edema (ml) | 0.907 | <0.001 | 0.922 | 0.747-0.976 | <0.001 |
| Gadollinium enhancement (gr) | 0.996 | <0.001 | 0.998 | 0.993-0.999 | <0.001 |
| **CI**: confidence interval**; ICC:** intraclass correlation coefficient **LV:** Left ventricle | | | | | |

**Table C. Intraobserver correlation and agreement**

| **VARIABLE** | **r** | **P** | **ICC** | **CI 95%** | **P** |
| --- | --- | --- | --- | --- | --- |
| End-diastolic volume (ml) | 0.966 | <0.001 | 0.987 | 0.958-0.996 | <0.001 |
| End-systolic volume (ml) | 0.966 | <0.001 | 0.981 | 0.940-0.994 | <0.001 |
| Ejection fraction (%) | 0.867 | <0.001 | 0.928 | 0.764-0.997 | <0.001 |
| LV mass (gr) | 0.951 | <0.001 | 0.974 | 0.916-0.992 | <0.001 |
| Stroke volume (ml) | 0.885 | <0.001 | 0.937 | 0.793-0.981 | <0.001 |
| LV edema (ml) | 0.988 | <0.001 | 0.991 | 0.969-0.997 | <0.001 |
| Gadollinium enhancement (gr) | 0.891 | <0.001 | 0.936 | 0.789-0.980 | <0.001 |
| **CI**: confidence interval**; ICC:** intraclass correlation coefficient **LV:** Left ventricle | | | | | |

**Table D. Baseline data in calcifediol and placebo groups in patients with FGF23 higher than the median at baseline**

| **VARIABLE** | **PLACEBO**  **N=8** | **CALCIFEDIOL**  **N=36** | **P** |
| --- | --- | --- | --- |
| **Clinical Data** |  |  |  |
| Age (years) | 55.0 (51.2, 58.5) | 63.0 (55.0, 70.8) | 0.051 |
| Gender (male, %) | 85.7 | 80.0 | 1.000 |
| Race (Caucasian, %) | 92.9 | 96.7 | 0.540 |
| Body Surface area (m^2^) | 1.88 (1.81, 1.98) | 1.91 (1.74, 2.06) | 0.948 |
| Body mass index (Kg/m^2^) | 25.8 (24.5, 29.4) | 27.3 (23.2, 29.7) | 0.675 |
| Diabetes (%) | 21.4 | 13.3 | 0.662 |
| Hypertension (%) | 42.9 | 33.3 | 0.783 |
| Smoker (%) | 71.4 | 53.3 | 0.419 |
| Dyslipidemia (%) | 64.3 | 43.3 | 0.332 |
| Culprit lesion |  |  | 0.358 |
| -Proximal LAD (%) | 42.9 | 36.7 |  |
| -Middle LAD (%) | 50.0 | 63.3 |  |
| -Distal LAD (%) | 7.1 | 0.0 |  |
| -First diagonal (%) | 0.0 | 0.0 |  |
| Number of vessels (%) |  |  | 0.355 |
| 1 | 92.9 | 73.3 |  |
| 2 | 7.1 | 20.0 |  |
| 3 | 0.0 | 6.7 |  |
| Number of vessels treated | 1.0 (1.0, 1.0) | 1.0 (1.0, 1.0) | 0.109 |
| Complete revascularization (%) | 92.9 | 86.7 | 1.000 |
| **Analytical Data** |  |  |  |
| eGFR (ml/min/1.73 m^2^) | 83.2 (73.4, 94.8) | 65.7 (59.6, 84.6) | **0.015** |
| Total cholesterol (mg/dl) | 172 (156, 194) | 186 (168, 200) | 0.736 |
| Low-density lipoprotein (mg/dl) | 118 (91, 137) | 114 (103, 137) | 0.939 |
| High density lipoprotein (mg/dl) | 35.0 (28.5, 41.2) | 40.0 (36.0, 45.0) | 0.198 |
| Triglycerides (mg/dl) | 143 (124, 190) | 115 (60, 154) | 0.110 |
| Hemoglobin (g/dl) | 14.3 (13.7, 14.6) | 14.6 (13.9, 16.0) | 0.189 |
| Glycemia (mg/dl) | 140 (124, 187) | 134 (112, 149) | 0.361 |
| Calcidiol (ng/ml) | 21.0 (19.0, 32.0) | 25.5 (17.0, 37.8) | 0.726 |
| Calcitriol (pg/mL) | 41.5 (34.2, 76.0) | 38.5 (28.2, 45.2) | 0.060 |
| FGF23 (RU/mL) | 100.0 (84.0, 124.0) | 106.0 (88.5, 150.0) | 0.420 |
| Klotho (pg/mL) | 706 (541, 905) | 560 (472, 710) | 0.085 |
| Parathormone (ng/mL) | 43.0 (36.0, 50.0) | 53.8 (34.0, 68.1) | 0.363 |
| Phosphate (mg/dl) | 4.3 (3.9, 4.8) | 4.2 (3.9, 5.0) | 0.964 |
| Calcium (mg/dL) | 9.6 (9.3, 10.0) | 9.7 (9.4, 10.0) | 0.683 |
| Hs-CRP (mg/L) | 31.0 (14.9, 54.6) | 13.6 (6.5, 41.1) | 0.071 |
| NT-proBNP (ng/L) | 1620 (766, 2560) | 1350 (646, 3012) | 0.721 |
| Galectin-3 (ng/mL) | 9761 (8539, 11122) | 9412 (7756, 12310) | 0.302 |
| MCP1 (pg/mL) | 134.0 (97.0, 172.0) | 124.0 (107.0, 145.0) | 0.801 |
| ST2 (pg/mL) | 19844 (16411, 31878) | 18369 (14548, 22711) | 0.326 |
| GDF15 (pg/mL) | 1066 (932, 1468) | 935 (690, 1748) | 0.456 |
| **Medication at discharge** |  |  |  |
| Acetylsalicylic acid (%) | 92.9 | 90.0 | 1.000 |
| P2Y12 inhibitor (%) | 92.9 | 100.0 | 0.318 |
| Statin (%) | 100.0 | 90.0 | 0.540 |
| Diuretic (%) | 35.7 | 33.3 | 1.000 |
| ARNI (%) | 0.0 | 0.0 | 1.000 |
| Betablocker (%) | 78.6 | 86.7 | 0.662 |
| ACEI / ARB (%) | 85.7 | 100.0 | 0.096 |
| MRA (%) | 28.6 | 50.0 | 0.313 |
| SGLT_2_ inhibitor (%) | 14.3 | 0.0 | 0.096 |
| Number of anti-remodeling drugs (%) |  |  | 0.090 |
| 0 | 7.1 | 0.0 |  |
| 1 | 21.4 | 6.7 |  |
| 2 | 35.7 | 50.0 |  |
| 3 | 28.6 | 43.3 |  |
| 4 | 7.1 | 0.0 |  |
| **Magnetic Resonance Imaging** |  |  |  |
| End-diastolic volume (ml) | 181 (135, 202) | 149 (109, 175) | 0.101 |
| End-diastolic volume by BSA (ml/m^2^) | 87.7 (73.0, 105.0) | 78.6 (62.3, 86.0) | 0.055 |
| End-systolic volume (ml) | 101.0 (78.9, 110.0) | 78.5 (55.5, 88.7) | 0.074 |
| End-systolic volume by BSA (ml/m^2^) | 49.6 (43.1, 58.6) | 40.1 (30.7, 46.0) | 0.052 |
| Ejection fraction (%) | 43.0 (40.6, 48.0) | 48.8 (42.5, 55.8) | 0.130 |
| LV mass (gr) | 113.0 (90.4, 127.0) | 120.0 (96.2, 135.0) | 0.909 |
| LV mass by BSA (gr/m^2^) | 57.7 (50.1, 64.6) | 60.6 (52.2, 65.8) | 0.861 |
| Stroke volume (ml) | 71.7 (56.4, 90.4) | 69.7 (59.0, 78.6) | 0.722 |
| Stroke volume by BSA (ml/m^2^) | 37.0 (31.0, 46.8) | 36.4 (32.4, 40.1) | 0.595 |
| LV edema (ml) | 32.7 (7.0, 42.1) | 29.8 (15.1, 43.9) | 0.666 |
| LV edema by BSA (ml/m^2^) | 18.1 (3.88, 24.2) | 15.1 (9.5, 21.6) | 0.623 |
| Gadollinium enhancement (gr) | 33.5 (23.7, 43.9) | 23.5 (11.5, 29.1) | **0.029** |
| Gadollinium enhancement by BSA (gr/m^2^) | 18.1 (13.6, 20.6) | 12.9 (7.33, 17.0) | **0.034** |

**ACEI:** Angiotensin-converting enzyme inhibitor; **ARB:** Angiotensin Receptor blocker; **ARNI:** Angiotensin receptor-neprilysin inhibitor; **BSA:** Body surface area; **eGFR:** estimated glomerular filtration rate; **FGF23:** Fibroblast growth factor-23; **GDF15:** Growth Differentiation Factor-15; **Hs-CRP:** High-sensitivity C-reactive protein; **LAD:** Left anterior descending coronary artery**; LV:** Left ventricle; **MCP-1:** Monocyte chemoattractant protein-1; **MRA:** Mineralcorticoid receptor antagonist; **NT-proBNP:** N-terminal pro-brain natriuretic peptide.

**Table E. Changes in Magnetic Resonance Imaging data in patients with FGF23 levels higher than the median at baseline**

| **VARIABLE** | **PLACEBO** | **CALCIFEDIOL** | **P** |
| --- | --- | --- | --- |
| End-diastolic volume (ml) | 7.7 (-24.0, 12.7) | 6.1 (-5.7, 20.5) | 0.841 |
| End-diastolic volume / BSA (ml/m^2^) | 4.4 (-11.0, 6.6) | 2.9 (-3.6, 11.2) | 0.806 |
| Percent increase in End-diastolic volume | 4.8 (-14.7, 8.1) | 4.0 (-3.8, 15.7) | 0.735 |
| End-systolic volume (ml) | -4.8 (-28.1, 17.6) | -7.0 (-13.2, 11.3) | 0.547 |
| End-systolic volume / BSA (ml/m^2^) | -2.8 (-14.5, 9.7) | -3.5 (-7.8, 5.4) | 0.503 |
| LV ejection fraction (%) | 9.6 (-2.0, 13.6) | 6.2 (0.1, 11.7) | 0.470 |
| LV mass (gr) | -11.3 (-15.8, -4.8) | -18.5 (-28.7, -4.2) | 0.302 |
| LV mass / BSA (gr/m^2^) | -5.6 (-8.8, -2.7) | -11.0 (-15.8, -2.1) | 0.329 |
| Stroke volume (ml) | 5.5 (-4.1, 24.5) | 10.3 (-0.4, 17.5) | 0.862 |
| Stroke volume / BSA (ml/m^2^) | 2.4 (-2.2, 13.6) | 5.4 (-0.4, 8.9) | 0.851 |
| LV edema (ml) | -24.0 (-42.1, -3.2) | -23.3 (-42.5, -10.6) | 0.777 |
| LV edema / BSA (ml/m^2^) | -11.7 (-24.0, -1.74) | -13.1 (-21.0, -5.6) | 0.750 |
| Gadollinium enhancement (gr) | -12.3 (-22.5, 2.1) | -5.0 (-11.7, -1.0) | 0.400 |
| Gadollinium enhancement / BSA (gr/m^2^) | -6.7 (-11.6, 1.00) | -2.7 (-6.3, -0.7) | 0.446 |

Changes are displayed as final-initial values. **Abbreviations: BSA:** Body surface area; **LV:** Left ventricle.

**Table F. Baseline data in calcifediol and placebo groups in patients with calcidiol lower than 30 ng/ml at baseline**

| **VARIABLE** | **PLACEBO** | **CALCIFEDIOL** | **P** |
| --- | --- | --- | --- |
| **Clinical Data** |  |  |  |
| Age (years) | 53.0 (51.0, 58.5) | 55.5 (48.8, 64.0) | 0.452 |
| Gender (male, %) | 94.4 | 87.5 | 0.655 |
| Race (Caucasian, %) | 94.4 | 92.5 | 0.538 |
| Body Surface area (m^2^) | 1.91 (1.82, 2.02) | 1.97 (1.84, 2.10) | 0.861 |
| Body mass index (Kg/m^2^) | 26.9 (26.1, 30.1) | 26.9 (24.7, 30.4) | 0.952 |
| Diabetes (%) | 11.1 | 17.5 | 0.706 |
| Hypertension (%) | 27.8 | 37.5 | 0.559 |
| Smoker (%) | 66.7 | 62.5 | 1.000 |
| Dyslipidemia (%) | 66.7 | 32.5 | **0.022** |
| Culprit lesion |  |  | 0.784 |
| -Proximal LAD (%) | 50.0 | 42.5 |  |
| -Middle LAD (%) | 50.0 | 52.5 |  |
| -Distal LAD (%) | 0.0 | 2.5 |  |
| -First diagonal (%) | 0.0 | 2.5 |  |
| Number of vessels (%) |  |  | 0.399 |
| 1 | 94.4 | 82.5 |  |
| 2 | 5.6 | 10.0 |  |
| 3 | 0.0 | 7.5 |  |
| Number of vessels treated | 1.0 (1.0, 1.0) | 1.0 (1.0, 1.0) | 0.246 |
| Complete revascularization (%) | 92.9 | 87.9 | 1.000 |
| **Analytical Data** |  |  |  |
| eGFR (ml/min/1.73 m^2^) | 84.9 (76.1, 96.9) | 82.9 (64.2, 93.1) | 0.437 |
| Total cholesterol (mg/dl) | 176 (156, 194) | 191 (178, 215) | 0.742 |
| Low-density lipoprotein (mg/dl) | 120 (91.2, 137) | 129 (107, 158) | 0.590 |
| High density lipoprotein (mg/dl) | 33.5 (27.2, 48.5) | 40.0 (33.0, 46.0) | 0.289 |
| Triglycerides (mg/dl) | 160 (84.0, 213) | 138 (106, 166) | 0.255 |
| Hemoglobin (g/dl) | 14.4 (14.1, 15.8) | 15.6 (14.6, 16.3) | 0.078 |
| Glycemia (mg/dl) | 134 (116, 158) | 134 (108, 165) | 0.937 |
| Calcidiol (ng/ml) | 19.0 (16.5, 23.8) | 17.0 (14.8, 23.5) | 0.362 |
| Calcitriol (pg/mL) | 41.5 (31.2, 73.2) | 38.5 (29.8, 50.0) | 0.255 |
| FGF23 (RU/mL) | 82.5 (64.2, 108.0) | 72.5 (53.8, 98.2) | 0.420 |
| Klotho (pg/mL) | 635 (512, 770) | 580 (486, 718) | 0.162 |
| Parathormone (ng/mL) | 43.0 (38.3, 50.0) | 45.7 (34.2, 52.3) | 0.666 |
| Phosphate (mg/dl) | 4.4 (3.9, 4.9) | 4.2 (3.5, 4.7) | 0.367 |
| Calcium (mg/dL) | 9.6 (9.4, 9.9) | 9.7 (9.3, 10.0) | 0.873 |
| Hs-CRP (mg/L) | 23.3 (12.4, 54.8) | 16.8 (7.78, 34.0) | 0.213 |
| NT-proBNP (ng/L) | 1400 (987, 1990) | 994 (544, 1980) | 0.086 |
| Galectin-3 (ng/mL) | 10204 (8164, 12670) | 9310 (7535, 10782) | 0.235 |
| MCP1 (pg/mL) | 125 (103, 143) | 130 (103, 152) | 0.827 |
| ST2 (pg/mL) | 20630 (16351, 29571) | 18802 (14329, 24370) | 0.565 |
| GDF15 (pg/ml) | 960 (681, 1211) | 824 (655, 1348) | 0.500 |
| **Medication at discharge** |  |  |  |
| Acetylsalicylic acid (%) | 94.4 | 95.0 | 1.000 |
| P2Y12 inhibitor (%) | 94.4 | 100.0 | 0.310 |
| Statin (%) | 100.0 | 92.5 | 0.545 |
| Diuretic (%) | 33.3 | 20.0 | 0.327 |
| ARNI (%) | 5.6 | 2.5 | 0.528 |
| Betablocker (%) | 83.3 | 87.5 | 0.694 |
| ACEI / ARB (%) | 88.9 | 95.0 | 0.581 |
| MRA (%) | 27.8 | 42.5 | 0.384 |
| SGLT_2_ inhibitor (%) | 11.1 | 2.5 | 0.225 |
| Number of anti-remodeling drugs (%) |  |  | 0.074 |
| 0 | 5.6 | 2.5 |  |
| 1 | 16.7 | 5.0 |  |
| 2 | 50.0 | 55.0 |  |
| 3 | 16.7 | 37.5 |  |
| 4 | 11.1 | 0.0 |  |
| **Magnetic Resonance Imaging** |  |  |  |
| End-diastolic volume (ml) | 176 (136, 211) | 159 (128, 192) | 0.205 |
| End-diastolic volume by BSA (ml/m^2^) | 88.7 (73.6, 102.0) | 79.2 (66.8, 90.4) | 0.125 |
| End-systolic volume (ml) | 95.0 (72.2, 111.0) | 80.0 (62.7, 93.2) | 0.311 |
| End-systolic volume by BSA (ml/m^2^) | 46.7 (35.5, 58.6) | 40.8 (32.0, 50.1) | 0.244 |
| Ejection fraction (%) | 47.1 (41.1, 53.6) | 47.7 (39.4, 53.9) | 0.746 |
| LV mass (gr) | 116 (109, 128) | 123 (102, 140) | 0.661 |
| LV mass by BSA (gr/m^2^) | 61.2 (52.6, 64.6) | 63.9 (55.3, 69.9) | 0.534 |
| Stroke volume (ml) | 80.5 (62.3, 92.9) | 73.3 (64.4, 81.2) | 0.336 |
| Stroke volume by BSA (ml/m^2^) | 41.0 (34.9, 46.1) | 38.0 (30.1, 41.0) | 0.225 |
| LV edema (ml) | 32.7 (5.66, 35.9) | 28.4 (16.0, 42.3) | 0.737 |
| LV edema by BSA (ml/m^2^) | 14.9 (2.6, 20.0) | 14.7 (8.5, 20.8) | 0.735 |
| Gadollinium enhancement (gr) | 30.1 (20.8, 43.9) | 25.9 (11.8, 33.6) | 0.050 |
| Gadollinium enhancement by BSA (gr/m^2^) | 17.8 (10.4, 20.6) | 12.7 (6.90, 17.3) | 0.059 |

**Abbreviations:** As for Table D

**Table G. Changes in Magnetic Resonance Imaging data in patients with calcidiol levels < 30 ng/ml**

| **VARIABLE** | **PLACEBO** | **CALCIFEDIOL** | **P** |
| --- | --- | --- | --- |
| End-diastolic volume (ml) | 6.1 (-2.5, 28.0) | 6.3 (-6.6, 19.3) | 0.915 |
| End-diastolic volume / BSA (ml/m^2^) | 3.6 (-1.3, 13.3) | 2.9 (-3.5, 10.0) | 0.979 |
| Percent increase in End-diastolic volume | 4.1 (-1.4, 11.4) | 3.7 (-3.7, 11.2) | 0.917 |
| End-systolic volume (ml) | 6.8 (-16.0, 18.1) | -8.9 (-18.4, 4.7) | 0.603 |
| End-systolic volume / BSA (ml/m^2^) | 3.9 (-8.1, 10.0) | -4.3 (-9.5, 3.3) | 0.722 |
| LV ejection fraction (%) | 1.1 (-2.6, 10.0) | 5.4 (2.9, 12.4) | 0.304 |
| LV mass (gr) | -13.3 (-20.5, -8.7) | -17.8 (-29.6, -5.2) | 0.286 |
| LV mass / BSA (gr/m^2^) | -6.6 (-11.0, -4.8) | -9.7 (-15.8, -2.6) | 0.286 |
| Stroke volume (ml) | 3.7 (-4.1, 12.5) | 11.5 (6.1, 17.8) | 0.264 |
| Stroke volume / BSA (ml/m^2^) | 2.0 (-2.2, 5.9) | 5.90 (3.0, 10.2) | 0.272 |
| LV edema (ml) | -20.5 (-35.8, -3.2) | -23.0 (-31.8, -7.6) | 0.773 |
| LV edema / BSA (ml/m^2^) | -10.2 (-20.0, -1.6) | -12.0 (-16.3, -3.6) | 0.743 |
| Gadollinium enhancement (gr) | -12.3 (-20.1, -0.9) | -5.0 (-13.1, -0.5) | 0.485 |
| Gadollinium enhancement / BSA (gr/m^2^) | -6.7 (-11.0, -0.5) | -2.7 (-7.3, -0.4) | 0.470 |

Changes are displayed as final-initial values. **Abbreviations: BSA:** Body surface area; **LV:** Left ventricle.

**Table H. Baseline data in calcifediol and placebo groups in patients with ejection fraction ≤40%**

| **VARIABLE** | **PLACEBO** | **CALCIFEDIOL** | **P** |
| --- | --- | --- | --- |
| **Clinical Data** |  |  |  |
| Age (years) | 48.5 (44.5, 55.8) | 53.0 (47.0, 56.0) | 0.599 |
| Gender (male, %) | 100.0 | 88.2 | 1.000 |
| Race (Caucasian, %) | 100.0 | 100.0 | 1.000 |
| Body Surface area (m^2^) | 1.96 (1.90, 2.01) | 2.00 (1.81, 2.21) | 0.705 |
| Body mass index (Kg/m^2^) | 24.8 (24.1, 26.6) | 29.4 (25.9, 31.4) | 0.194 |
| Diabetes (%) | 25.0 | 17.6 | 1.000 |
| Hypertension (%) | 50.0 | 29.4 | 0.394 |
| Smoker (%) | 62.5 | 76.5 | 0.640 |
| Dyslipidemia (%) | 50.0 | 29.4 | 0.394 |
| Culprit lesion |  |  | 1.000 |
| -Proximal LAD (%) | 57.1 | 50.0 |  |
| -Middle LAD (%) | 42.9 | 50.0 |  |
| -Distal LAD (%) | 0.0 | 0.0 |  |
| -First diagonal (%) | 0.0 | 0.0 |  |
| Number of vessels (%) |  |  | 1.000 |
| 1 | 87.5 | 88.2 |  |
| 2 | 12.5 | 11.8 |  |
| 3 | 0.0 | 0.0 |  |
| Number of vessels treated | 1.0 (1.0, 1.0) | 1.0 (1.0, 1.0) | 0.145 |
| Complete revascularization (%) | 87.5 | 88.2 | 1.000 |
| **Analytical Data** |  |  |  |
| eGFR (ml/min/1.73 m^2^) | 77.1 (67.5, 91.4) | 89.1 (77.7, 95.5) | 0.374 |
| Total cholesterol (mg/dl) | 180 (160, 260) | 179 (168, 192) | 0.838 |
| Low-density lipoprotein (mg/dl) | 105 (69.0, 184) | 109 (102, 130) | 0.928 |
| High density lipoprotein (mg/dl) | 40.0 (31.8, 57.5) | 34.0 (30.8, 42.2) | 0.425 |
| Triglycerides (mg/dl) | 124 (89, 240) | 142 (120, 182) | 0.953 |
| Hemoglobin (g/dl) | 14.6 (14.2, 15.7) | 14.7 (13.9, 16.1) | 0.951 |
| Glycemia (mg/dl) | 121 (104, 148) | 130 (116, 144) | 0.444 |
| Calcidiol (ng/ml) | 27.5 (18.0, 38.8) | 19.5 (16.8, 32.5) | 0.443 |
| Calcitriol (pg/mL) | 72.0 (48.8, 90.5) | 47.0 (39.8, 69.2) | 0.153 |
| FGF23 (RU/mL) | 80.0 (63.2, 85.2) | 67.5 (45.8, 136.0) | 0.951 |
| Klotho (pg/mL) | 546 (493, 713) | 549 (473, 617) | 0.783 |
| Parathormone (ng/mL) | 37.3 (33.6, 44.0) | 34.7 (27.7, 49.7) | 0.759 |
| Phosphate (mg/dl) | 4.70 (3.80, 4.85) | 4.00 (3.75, 5.05) | 0.972 |
| Calcium (mg/dL) | 9.8 (9.6, 9.9) | 9.5 (9.00, 9.9) | 0.300 |
| Hs-CRP (mg/L) | 56.7 (26.9, 158.0) | 25.6 (13.1, 59.6) | 0.214 |
| NT-proBNP (ng/L) | 1060 (982, 1860) | 1120 (750, 2048) | 0.867 |
| Galectin-3 (ng/mL) | 11362 (9574, 13930) | 9218 (6820, 9815) | **0.027** |
| MCP1 (pg/mL) | 114.0 (94.2, 134.0) | 124.0 (104.0, 147.0) | 0.616 |
| ST2 (pg/mL) | 28811 (23883, 37303) | 21477 (16263, 31631) | 0.341 |
| GDF15 (pg/ml) | 792 (705, 1810) | 826 (690, 1070) | 0.713 |
| **Medication at discharge** |  |  |  |
| Acetylsalicylic acid (%) | 100.0 | 100.0 | 1.000 |
| P2Y12 inhibitor (%) | 100.0 | 100.0 | 1.000 |
| Statin (%) | 100.0 | 94.1 | 1.000 |
| Diuretic (%) | 37.5 | 29.4 | 1.000 |
| ARNI (%) | 12.5 | 0.0 | 0.320 |
| Betablocker (%) | 100 | 94.1 | 1.000 |
| ACEI / ARB (%) | 75.0 | 82.4 | 1.000 |
| MRA (%) | 37.5 | 70.6 | 0.194 |
| SGLT_2_ inhibitor (%) | 12.5 | 5.9 | 1.000 |
| Number of anti-remodeling drugs (%) |  |  | 0.154 |
| 0 | 0.0 | 0.0 |  |
| 1 | 12.5 | 11.8 |  |
| 2 | 50.0 | 23.5 |  |
| 3 | 25.0 | 64.7 |  |
| 4 | 12.5 | 0.0 |  |
| **Magnetic Resonance Imaging** |  |  |  |
| End-diastolic volume (ml) | 182 (180, 193) | 197 (140, 214) | 0.662 |
| End-diastolic volume by BSA (ml/m^2^) | 94.6 (89.0, 101.0) | 90.3 (74.4, 106.0) | 0.842 |
| End-systolic volume (ml) | 110.0 (108.0, 129.0) | 125.0 (84.3, 141.0) | 0.977 |
| End-systolic volume by BSA (ml/m^2^) | 57.1 (54.0, 65.6) | 56.6 (51.5, 67.2) | 0.754 |
| Ejection fraction (%) | 37.8 (30.4, 39.9) | 37.4 (34.2, 39.1) | 0.930 |
| LV mass (gr) | 141 (124, 154) | 123 (108, 136) | 0.322 |
| LV mass by BSA (gr/m^2^) | 71.4 (63.5, 77.2) | 64.6 (58.0, 68.5) | 0.402 |
| Stroke volume (ml) | 70.4 (60.0, 72.8) | 65.4 (57.0, 77.8) | 0.884 |
| Stroke volume by BSA (ml/m^2^) | 35.0 (31.3, 36.6) | 32.4 (28.4, 37.1) | 0.588 |
| LV edema (ml) | 29.6 (17.8, 38.7) | 37.2 (20.1, 58.0) | 0.333 |
| LV edema by BSA (ml/m^2^) | 14.7 (9.35, 19.7) | 20.8 (10.1, 25.2) | 0.401 |
| Gadollinium enhancement (gr) | 34.1 (26.8, 42.8) | 26.8 (24.1, 39.4) | 0.811 |
| Gadollinium enhancement by BSA (gr/m^2^) | 16.9 (13.4, 21.9) | 16.2 (12.7, 20.7) | 0.868 |

**Abbreviations:** As for Table D

**Table I. Changes in Magnetic Resonance Imaging data in patients with ejection fraction ≤40% at baseline**

| **VARIABLE** | **PLACEBO** | **CALCIFEDIOL** | **P** |
| --- | --- | --- | --- |
| End-diastolic volume (ml) | 8.65 (-6.43, 40.6) | -1.35 (-6.70, 22.4) | 0.511 |
| End-diastolic volume / BSA (ml/m^2^) | 4.53 (-3.30, 20.0) | -0.68 (-3.40, 12.4) | 0.588 |
| Percent increase in End-diastolic volume | 4.75 (-3.53, 20.4) | -0.62 (-3.22, 16.6) | 0.549 |
| End-systolic volume (ml) | 3.98 (-33.3, 26.4) | -11.3 (-26.1, 4.54) | 0.44 |
| End-systolic volume / BSA (ml/m^2^) | 2.49 (-17.0, 13.0) | -5.00 (-17.4, 2.26) | 0.549 |
| LV ejection fraction (%) | 11.0 (6.36, 18.3) | 6.11 (3.80, 12.6) | 0.44 |
| LV mass (gr) | -37.7 (-42.3, -15.8) | -11.3 (-28.0, -3.98) | 0.188 |
| LV mass / BSA (gr/m^2^) | -19.2 (-21.3, -8.73) | -5.58 (-15.0, -1.96) | 0.188 |
| Stroke volume (ml) | 20.0 (15.6, 30.1) | 12.0 (8.41, 17.9) | 0.124 |
| Stroke volume / BSA (ml/m^2^) | 9.9 (7.81, 15.6) | 5.99 (4.16, 9.7) | 0.238 |
| LV edema (ml) | -20.4 (-30.7, -10.8) | -26.1 (-47.0, -12.5) | 0.392 |
| LV edema / BSA (ml/m^2^) | -10.2 (-15.4, -5.69) | -15.6 (-20.3, -6.30) | 0.428 |
| Gadollinium enhancement (gr) | -10.8 (-20.6, -4.97) | -6.46 (-24.4, -0.16) | 0.973 |
| Gadollinium enhancement / BSA (gr/m^2^) | -5.40 (-11.2, -2.63) | -3.80 (-11.3, -0.08) | 0.815 |

Changes are displayed as final-initial values. **Abbreviations: BSA:** Body surface area; **LV:** Left ventricle.
